# The feasibility of using smartphones and mobile breathalyzers to monitor alcohol consumption among people living with HIV/AIDS

**DOI:** 10.1186/s13722-019-0174-0

**Published:** 2019-11-26

**Authors:** Carolyn Lauckner, Erica Taylor, Darshti Patel, Alexis Whitmire

**Affiliations:** Department of Health Promotion and Behavior, University of Georgia, 321B Wright Hall, 100 Foster Rd, Athens, GA 30602 USA

**Keywords:** Alcohol use, mHealth, Electronic momentary assessment, HIV/AIDS

## Abstract

**Background:**

For people living with HIV/AIDS (PLWHA), alcohol consumption is associated with poor treatment outcomes and medication adherence. This pilot study examined the feasibility of using smartphones and mobile Bluetooth breathalyzers for monitoring alcohol consumption among PLWHA (*N* = 17).

**Methods:**

For 2 weeks, participants responded to twice-daily text message prompts by completing a breathalyzer reading and a mobile survey about their alcohol use. They also completed baseline questionnaires assessing alcohol consumption and hazardous drinking behaviors.

**Results:**

Participants completed an average of 22 of 28 breathalyzer readings and 17 of 28 mobile surveys, and were more likely to complete daytime (vs. evening) monitoring tasks. Results suggested that self-reported frequency of binge drinking at baseline was related to an increased number of days with alcohol consumption according to breathalyzer and mobile surveys, as well as a higher average blood alcohol content. Qualitative interviews found generally positive attitudes toward the technologies, but some participants reported experiencing technical difficulties.

**Conclusions:**

Overall, this preliminary research suggests that smartphone monitoring of alcohol consumption among PLWHA may reflect cross-sectional self-reported alcohol consumption behaviors, but could use improvements to increase adherence to monitoring tasks.

## Background

People living with HIV/AIDS (PLWHA) are almost twice as likely to use alcohol as those in the general population [1], and as many as half of PLWHA have a history of alcohol-related problems [2]. Alcohol can have severe negative effects on PLWHA by way of several mechanisms, but it has an especially negative impact on antiretroviral therapy (ART) adherence. On days when individuals have one or more drinks of alcohol, they are almost nine times more likely to be non-adherent to their medication regimen [3]. One study found that alcohol consumption was the strongest predictor of ART adherence, having larger effects than depression, social support, heroin use, cocaine use, dosage amount, age, gender, or race/ethnicity [4]. It is clear that alcohol consumption must be addressed to increase ART adherence and survival among PLWHA.

The strong link between alcohol consumption and treatment use and outcomes has led to interventions to reduce alcohol use among PLWHA, using methods including motivational interviewing and cognitive behavioral therapy. These interventions have had mixed results, with some leading to increases in medication use [5] and decreases in alcohol consumption [6], while others have not had any significant effects [7]. A recent study by Satre et al. [8] found that a motivational interviewing intervention for PLWHA compared to treatment as usual did not have any overall effects on alcohol consumption, but did have a significant effect among those who had low motivation to reduce their alcohol use. Importantly, a meta-analysis of alcohol interventions for PLWHA found that behavioral interventions have been successful overall at reducing quantity of alcohol consumed and heavy drinking, with effect sizes ranging from *d* = 0.11 to 0.24 [9]. This study also found that 91% of these interventions were conducted in-person [9]. This finding, coupled with research suggesting that even brief interventions are more effective if delivered over multiple sessions [10], suggests that current models of alcohol reduction interventions may not be accessible to individuals who cannot easily attend in-person appointments. This is concerning, given that over 40% of HIV case managers report that a lack of transportation is a “major problem” for their clients [11]. Researchers have suggested that technology may be a useful tool for further increasing access to these interventions, particularly among those living in rural areas who cannot easily access treatment facilities [7].

Hasin et al. [12] tested a brief intervention among PLWHA using motivational interviewing combined with daily self-monitoring of alcohol consumption using telephone-based interactive voice response. The addition of phone-based monitoring led to decreases in consumption among problem drinkers as compared to control and motivational interviewing-only conditions. Follow-up research tested a smartphone version of the daily self-monitoring and found that the app had higher participation and retention rates than the interactive voice response version, and when tested in a randomized controlled trial it led to a significant decrease in drinking days compared to motivational interviewing-only [13].

Other researchers have started to utilize this smartphone-based approach, using these devices to conduct ecological momentary assessment (EMA) of alcohol use for PLWHA. EMA provides a framework of frequent data collection, serving as an essential tool for documenting within-person changes. It also reduces the risk of inaccurate recall found in other forms of self-report and acknowledges the dynamic nature of drinking habits [14]. Moore et al. [15] found that smartphones proved to be a feasible and an acceptable EMA method among an older HIV+ adult group. Within this study, 60% of participants reported that the smartphone did not interfere with their daily activities, while the EMA adherence percentage per person was 86.4%. Similarly, Paolillo et al. [16] found high EMA adherence rates (89.5%) and agreement that the questions did not interfere with daily life. Another study assessing the feasibility and acceptability of smartphone apps for daily reporting of substance use and ART adherence found that there were high completion rates and participant acceptability in addition to a high satisfaction outcome with using these smartphone apps as a tracking method [17]. These studies suggest that these smartphone apps can be feasible for data collection, and research also suggests that they may be valid measures of substance use. Studies using traditional recall-based methods of self-report and laboratory measurements of alcohol use have found correlations between these outcome measures and EMA data [16, 18].

The tracking and reducing alcohol consumption (TRAC) pilot study built on this research by utilizing smartphones and EMA paired with mobile Bluetooth breathalyzers measuring blood alcohol content (BAC) to monitor alcohol consumption among PLWHA for a 2-week period. This approach sought to examine the feasibility of gathering behavioral data on a twice-daily basis with this population. To date, EMA approaches have rarely been combined with BAC assessment, which has potential to improve the quality of alcohol consumption data. Morgenstern et al. [14], in a review of alcohol-based EMA, suggested that combining EMA self-report data with biological sensor-based data such as that collected from breathalyzers holds “promise to significantly improve investigators’ ability to accurately assess alcohol consumption, understand the determinants of risky drinking, and trigger real-time interventions” (p. 102). Thus, in this feasibility study, we tested the feasibility and preliminary validity of this form of EMA by examining the following research questions (RQs):

- RQ1: How adherent are PLWHA to twice-daily monitoring of alcohol consumption?
- RQ2: Is there a relationship between adherence to twice-daily monitoring and baseline self-reported alcohol consumption behaviors?
- RQ3: What is the frequency of alcohol consumption among PLWHA according to twice-daily monitoring?
- RQ4: What is the relationship between baseline self-reported alcohol consumption and alcohol consumption as measured by twice-daily monitoring?
- RQ5: What qualitative feedback did the participants provide regarding the technology used in the TRAC pilot study?

We opted to do twice-daily monitoring at two random time points based on a review of the previous literature. A previous study required participants to complete phone based alcohol self-monitoring at four random points each day and reported 97% compliance [19]. Another study asked individuals with sexually transmitted infections to complete surveys on cell phones three times daily over 12 weeks and found that not only did 88% complete the entire 12-week study, but that these individuals completed 90% of the requested surveys [20]. Because of the added burden of completing a breathalyzer reading in addition to a mobile survey, we aimed for two monitoring tasks/day in order to optimize compliance. Given that morning or daytime drinking is an indicator of alcohol abuse or dependence according to several validated scales [21, 22], we included a daytime monitoring task when possible to potentially capture these behaviors.

## Methods

### Study overview

During 2017 to 2018, the TRAC study asked participants to monitor their alcohol consumption over a 2-week period using mobile phone technologies. Each participant was provided with a smartphone and a mobile Bluetooth-enabled breathalyzer, a BACTrack Mobile Pro. The smartphone was pre-loaded with the BACTrack app and also contained a shortcut to a mobile survey assessing alcohol use. For this 2-week period, participants were texted at two random times each day (in the daytime, typically before 5 p.m., and in the evening) and asked to complete a breathalyzer reading and mobile survey. The BACTrack app guided participants through the process of completing a breathalyzer reading on their Bluetooth-enabled device and allowed them to send the reading directly to the researchers. Participants also completed a short baseline questionnaire regarding their health behaviors and a qualitative exit interview regarding their experiences with the study.

### Participants

Participants in the study consisted of PLWHA in a non-metropolitan setting in the Southern region of the United States. Eligible and enrolled participants (*N* = 20) were all currently on medication for HIV/AIDS, over 21 years old, and had at least one alcoholic drink per week. Participants were recruited through flyers about the study that were posted at local clinics and were also referred to the study by clinic case managers. Because recruiting was done using these passive methods in combination with referrals, the recruitment success rate cannot be calculated. Two participants were completely non-adherent to the monitoring tasks, and one participant’s data were lost during the data collection process, so this manuscript reports primarily on the 17 participants who completed at least one monitoring task. Participants’ ages ranged from 26 to 60, with a mean of 47.5 years. Sixty-five percent of participants were male and 35% identified as female. See Table 1 for full demographic details and baseline characteristics.

**Table 1:** Participant demographics and baseline characteristics (N = 20)

Average age	47.35 (± 9.56)
Sexual orientation	
Gay/homosexual	8 (40%)
Bisexual	1 (5%)
Straight/heterosexual	9 (45%)
Other	2 (10%)
Gender	
Male	13 (65%)
Female	7 (35%)
Race	
Black/African American	14 (70%)
American Indian or Alaskan Native	2 (10%)
White	6 (30%)
Highest level of education attained	
Some high school	6 (30%)
High school degree	1 (5%)
Some college	10 (50%)
College degree	2 (10%)
Graduate degree	1 (5%)
Income	
$0–10,000	11 (55%)
$11–20,000	5 (25%)
$21–30,000	3 (15%)
$31–40,000	1 (5%)
Employment status	
Working	4 (20%)
Disability	11 (55%)
Unemployed	5 (25%)
Current relationship status	
Not having sex, no partner/significant other	9 (45%)
Having sex, but with more than one partner	2 (10%)
Having sex with just one partner, but for less than 6 months	1 (5%)
Having sex with just one partner for 6 months or longer	8 (40%)
Average time since diagnosis	13.82 years (± 9.28)
Medication adherence	
What percent of medication taken in the past month	85.83 (±24.69)
Average missed doses in the past week	0.47 (± 1.81)
Alcohol use	
AUDIT score	11.12 (± 8.31)
Average days participant consumed alcohol in past month	10.32 (± 8.54)
Average days participant had five or more drinks in past month	3.95 (± 7.48)

### Procedures

After eligibility screening, participants were enrolled in the study and scheduled for a baseline appointment. Upon arrival, informed consent was obtained from all individual participants included in the study. Participants first completed a survey about their current alcohol use, medication adherence, and demographics. They were then shown how to use the smartphone, BACTrack breathalyzer, BACTrack app, and mobile survey. After the tutorial concluded and participants practiced using the technologies, they were given the equipment and instructions for completing the monitoring tasks. During the next 2 weeks, participants were prompted twice daily by text message (for a total of 28 prompts) to complete a breathalyzer reading, share the reading with the researchers, and complete a mobile questionnaire about their alcohol use. The participants used the BACTrack app to forward the readings automatically to the research coordinator. After participants hit a “Share” button within the app, the app generated a text message with the BAC level along with a link for viewing the result.

A research assistant randomly chose times for the participants to receive prompts that were delivered using online text messaging software, with the earliest of the daytime texts being sent at 6 a.m. and the latest sent at midnight based on the participants’ predetermined “do not disturb” hours. These hours were times when they would be unable or unwilling to complete a reading, such as if they were going to be at work or school. It should be noted that while we call the first monitoring task of the day the “daytime” task, there were two participants who indicated that they could not complete any monitoring tasks during the daytime (i.e., before 5 p.m.), typically due to work requirements. These individuals did receive two prompts, but they were both after the 5 p.m. hour, with one individual who worked a night shift typically getting their first reminder around 10 or 11 p.m. and their second around 5 or 6 a.m. Participants did not receive text message prompts at the same time 2 days in a row.

The surveys required a mobile data connection, so participants were given study phones to use in order to ensure a consistent data connection. Following the monitoring period, participants were asked to come in for a final appointment, where they returned their equipment, completed the same survey that they filled out at baseline, and were interviewed about their perceptions of the study. The participation incentives for the study varied, and the most that participants could receive was $130. The participants were paid at the first interview, last interview, and for completing the daily monitoring. Specifically, for each day that participants completed both daytime and evening monitoring tasks (sending in both a breathalyzer reading and mobile questionnaire each time), they received $5. All procedures were approved by the University’s Institutional Review Board. While we assessed multiple variables of interest through the various forms of data collection, this manuscript focuses specifically on the alcohol consumption data collected via the baseline survey and the mobile monitoring tasks, as well as the qualitative exit interview.

### Quantitative measures

Adherence to monitoring was calculated by adding up the total number of breathalyzer readings reported to the researchers and mobile surveys completed. The mobile surveys contained up to 11 questions, with participants completing fewer if they had not consumed alcohol that day. If they reported drinking (yes/no), they were asked how many drinks they had consumed, how long it had been since their last drink (in minutes), and if they were planning to drink other alcohol that day (yes/no).

As an indicator of daily alcohol consumption throughout the monitoring period, we examined how often participants reported having consumed alcohol (yes/no) in their surveys and added up the total instances in which breathalyzer readings were higher than 0.000. We then divided these numbers by the total number of breathalyzer readings/surveys returned per participant in order to obtain an estimated percentage of drinking instances during the monitoring period. We also calculated these numbers separately for daytime and evening monitoring tasks.

Problematic alcohol consumption was measured at baseline using the alcohol use disorders identification test (AUDIT) [21], a 10-item scale aimed to identify patterns of hazardous or harmful drinking. Items include “How often do you have a drink containing alcohol?”, “How often do you have six or more drinks on one occasion?”, and “How many drinks containing alcohol do you have on a typical day when you are drinking?”, among others. Responses were assigned values ranging from 0 to 4, and the total values were summed. Participants were also asked to report how many days they drank in the past month and how many days they had five or more drinks of alcohol.

### Qualitative measures

Participant feedback regarding the study was solicited using a semi-structured interview protocol that focused on their entry into the study, their opinions toward using the BACTrack breathalyzer, the iPhone, and the BACTrack app; their initial reactions to the study; and their perceived usefulness of the technology and alcohol consumption tracking. These interviews were done face-to-face, audio recorded, and transcribed. The first section focused on the participants’ feelings about the study enrollment process and items included questions such as, “How were you recruited for this study?” The second section focused on the participant’s initial feelings prior to starting the study and items included questions such as, “Why did you decide to participate in the study?” and “Tell me about your initial reaction to the TRAC study and the monitoring you were asked to do.” The next section was focused on the participant’s experience with the technology and these items included questions such as “How was your overall experience using the smartphone?” and “Was there anything challenging with using the technology?” The following section was focused specifically on the breathalyzer, including questions such as “How comfortable did you feel using the breathalyzer technology” and “Was the breathalyzer easy or difficult to use?” The final section focused on the participant’s overall reflection on their involvement in the study and the items included questions such as, “What suggestions do you have for improving the TRAC Study,” and “Do you think the use of smartphones is a good way to monitor alcohol consumption?,” and “Do you think it was worthwhile to participate in TRAC?” Due to the semi-structured format, all participants may not have received exactly the same questions, but the same topics were touched on by the interviewers with all participants. Because we wanted to capture the experiences of all participants enrolled in the study, the sample size for the interviews was N = 20 (including the two non-adherent participants and the participant whose monitoring data were lost).

### Analysis

To examine RQ1, which concerned adherence to monitoring tasks, we calculated percentages of breathalyzer readings and mobile surveys completed out of 28 possible. To determine if there were differences in the numbers of breathalyzer and mobile surveys completed for the daytime vs. the evening monitoring tasks, we conducted paired samples t-tests. RQ2 was explored by calculating correlations between baseline measures of alcohol use (AUDIT score, drinking in the past month, binge drinking in the past month) and number of missing breathalyzer readings and mobile surveys. We examined RQ3 by calculating the frequency of occurrences in which participants had positive breathalyzer readings (i.e., above 0.000) and reported consuming alcohol in the survey, as well as by examining average BAC scores and number of drinks reported in the surveys. We also examined differences between breathalyzer and mobile survey reports of alcohol consumption by counting the instances in which the breathalyzer reading and mobile survey response matched in terms of whether or not they indicated alcohol consumption.

To examine RQ3, we conducted one-tailed correlation analyses between baseline levels of alcohol consumption and the following data points generated by the twice-daily monitoring: the average BAC reported for each participant across their multiple breathalyzer readings, the percentage of days the participants drank calculated based on days in which there were available breathalyzer readings, and percentage of days the participants drank calculated based on days in which there were available mobile surveys. One-tailed analyses were conducted because there was a clear, expected direction of the relationships observed, such that higher alcohol consumption at baseline would be associated with higher alcohol consumption during the monitoring period.

Quantitative coding procedures were used to examine RQ4. A research team consisting of the PI and four research assistants reviewed the transcripts and developed a coding scheme based on the responses generated, using a grounded approach [23]. This coding scheme was developed according to each question in the interview protocol, with unique codes developed for responses to each question. Because not all participants were asked exactly the same questions, in some cases the sample size for the coding was less than N = 20. Each transcript was coded to agreement by two members of the research team, with percent agreement rates ranging from 80 to 100%. If discrepancies remained after adequate percent agreement was obtained, the research team discussed the responses until a consensus was reached regarding the appropriate code. Frequencies for each code were then generated and representative quotations were pulled from transcripts to provide examples of themes.

## Results

### Daily monitoring results (RQ1)

Most of the results that follow are based on 17 participants who participated in the twice-daily monitoring; however, there were 20 total participants enrolled in the study that completed baseline and post-test surveys. Two of these participants were entirely non-adherent and did not complete any of the breathalyzer readings or mobile surveys, and one participant’s data was lost during the research process. The two non-adherent participants were followed up with in their final interview. When these participants were asked about their feelings toward the cell phone and breathalyzer during the final interview some of the responses included, “it was simple when we did it in here, it was fine. But then not being able to use the smart phone out there…I got aggravated,” and “Well I’m not used to using smart phones and I didn’t know nothing about the breathalyzer.”

Table 2 contains descriptive statistics for adherence to monitoring among the 17 participants who completed at least one survey or breathalyzer reading. Overall, participants completed more breathalyzer readings (80% of all possible) than mobile surveys (62%). On average, participants completed at least one breathalyzer reading on 89% of the days and at least one survey on 77% of the days. Three participants completed 100% of the breathalyzer readings and two participants completed 100% of the mobile surveys. They completed significantly more daytime breathalyzer readings than evening breathalyzer readings (*t* (16) = 2.89, *p* < 0.05, *d* = 0.70), as well as more daytime mobile surveys than evening mobile surveys (*t* (16) = 4.06, *p* = 0.001, *d* = 0.98). Nine participants completed both breathalyzer readings on the final day of the monitoring period (n = 16 completed at least one), and nine participants completed both surveys on the final day (n = 13 completed at least one).

**Table 2:** Monitoring adherence and results (*n* = 17)

	% or M(SD)
Breathalyzer readings	
Number of breathalyzer readings (out of 28)	22.29 (5.65)
Days with at least one breathalyzer reading (out of 14)	12.47 (2.12)
Number of daytime breathalyzer readings (out of 14)	12.06 (2.14)
Number of evening breathalyzer readings (out of 14)	10.24 (3.85)
Average BAC level	0.02 (0.03)
Mobile surveys	
Number of mobile surveys (out of 28)	17.41 (8.52)
Days with at least one mobile survey (out of 14)	10.71 (4.21)
Number of daytime mobile surveys (out of 14)	10.29 (4.21)
Number of evening mobile surveys (out of 14)	7.12 (4.87)
Average number of drinks reported/survey	2.62 (2.09)
Alcohol use	
Average percent of total breathalyzer readings indicating alcohol consumption^a^	20%
Average percent of daytime breathalyzer readings indicating alcohol consumption^a^	19%
Average percent of evening breathalyzer readings indicating alcohol consumption^a^	22%
Average percent of total surveys indicating alcohol consumption^a^	25%
Average percent of daytime surveys indicating alcohol consumption^a^	20%
Average percent of evening surveys indicating alcohol consumption^a^	40%
Average percent of days with at least one breathalyzer reading indicating alcohol consumption^a^	23%
Average percent of days with at least one survey indicating alcohol consumption^a^	34%
Average percent of time that breathalyzer and survey matched in terms of detecting alcohol consumption	90%

^a^Percentage is calculated based on number of readings/surveys submitted

If taking into account the two participants who did not complete any monitoring tasks, the participants completed an average of 71% of readings and 56% of surveys. The average percentage of days with at least one breathalyzer reading was 80% and days with at least one survey was 68%.

### Correlation between adherence and baseline alcohol consumption (RQ2)

Correlation analyses were conducted to examine if adherence to the mobile monitoring was associated with higher levels of alcohol consumption at baseline. There were no significant correlations between number of breathalyzer readings and mobile surveys completed and baseline measures of binge drinking, days the participants drank alcohol, or AUDIT scores.

### Daily reports of alcohol consumption (RQ3)

Table 2 contains statistics regarding the alcohol consumption reported by participants through the twice-daily monitoring. While the overall number of times in which participants reported drinking alcohol was low, after adjusting for the total number of monitoring tasks completed, over 20% of the reports indicated that participants had been drinking. Participants had higher frequencies of positive breathalyzer readings and surveys indicating drinking for the evening tasks compared to the daytime tasks. Overall, the mobile surveys yielded higher rates of reported alcohol consumption than the breathalyzer readings. If considering alcohol consumption on a day-by-day basis, participants drank on 23% of the monitoring days according to breathalyzer readings and on 34% of monitoring days according to mobile surveys, adjusted for the number of reports submitted. The average BAC level for participants ranged from 0 to 0.09, while the average number of drinks reported by participants ranged from 0 to 6.75.

Out of the 278 times that all the participants completed both a breathalyzer and a mobile survey reading, in 90% of cases (*n* = 249) both the breathalyzer reading and the mobile survey matched in terms of whether they reflected alcohol consumption. Among the cases that did not match, 72% were times when the participants reported consuming alcohol in the survey but did not have a positive breathalyzer reading. In these instances, the time reported since the last drink ranged from 10 min to 15 h, with the average being 4 h and 28 min. The average number of drinks consumed according to the surveys ranged from 1 to 3, with the average being 1.26 drinks.

### Relationship between baseline self-reported behaviors and behaviors captured via monitoring (RQ4***)***

Correlation analyses were conducted to examine how baseline alcohol consumption behaviors were related to the behaviors observed during the monitoring period (see full results in Table 3). A significant relationship was observed between participants’ AUDIT scores and their average BAC level across the monitoring period, *r* = 0.52, *p* < 0.05. There were also significant relationships observed between the frequency of binge drinking at baseline and the following variables: percentage of days participants drank based on breathalyzers (*r* = 0.55, *p* < 0.05), percentage of days participants drank based on mobile surveys (*r* = 0.63, *p* < 0.05), and average BAC level (*r* = 0.61, *p* < 0.01). There were no significant relationships observed between the number of days participants drank in the past month and any of the alcohol-related monitoring variables.

**Table 3:** Correlations between baseline and monitoring-based alcohol consumption behaviors

		1	2	3	4	5	6
1	AUDIT (BL)	–					
2	Binge drinking frequency (BL)	0.75**	–				
3	Alcohol consumption frequency (BL)	0.79**	0.52*	–			
4	Percentage of drinking days (breathalyzer)	0.35	0.55^*^	0.15	–		
5	Percentage of drinking days (survey)	0.43	0.63*	0.21	0.92***	–	
6	Average BAC (breathalyzer)	0.52*	0.61**	0.26	0.95***	0.86***	–

***Correlation is significant at the 0.001 level (1-tailed)

**Correlation is significant at the 0.01 level (1-tailed)

*Correlation is significant at the 0.05 level (2-tailed)

### Qualitative feedback on TRAC pilot study (RQ4)

#### Overall opinions on monitoring

Participants were asked if they believed the monitoring seemed useful overall. A strong majority of the participants (16/20, 80%) responded that they generally believed the monitoring was useful or were more specific, stating that it was useful because it made them want to drink less or at least think about their drinking. For example, one participant stated: “I thought it was interesting. You know, it makes me think about what I'm drinking, how much I'm drinking, you know that kind of thing. It makes you think. Eventually I am going to try to stop drinking.”

This was echoed in the responses to the question regarding their overall experience in tracking their alcohol consumption. Five participants (25%) reported that it made them more aware of their drinking behavior or change their drinking behaviors, such as the participant who stated, “It actually made me slow down because I didn’t want to send in [a high reading].” Two participants (10%) stated that completing the alcohol tracking was easy to do, while another two participants stated that they would have changed something about the way the monitoring was done. One participant, for example, suggested that we included follow-up prompts if a high reading was obtained: “Say if you get a higher reading maybe there would be more questions to figure out if there was a reason why it was higher.”

We also asked if they thought smartphones could be a good tool in helping to reduce alcohol consumption. One person stated that they did not think so, and three participants (15%) said they could be a good tool but only with improvements to the technology or more user training. One of these individuals stated “A little more practice and a little bit more confidence in using it…I think it would work out better for me.” Three participants (15%) noted that mobile monitoring could help to increase awareness of drinking or sobriety level—such as the individual who said “Yeah, because I don’t think you really realize how much you are drinking, you know?” Four individuals (20%) specifically stated that they could be helpful tools to help individuals in specific high-risk situations, such as those looking to avoid drinking and driving, individuals who are alcohol dependent, or those who are on probation. For example, one participant said “They need to give to people that get DUIs, you know, they need to be blowing before they get in the car. That would save a lot of lives, I do believe.” An additional six participants (30%) agreed that smartphones could be good tools for reducing alcohol consumption, either in general terms or for other reasons not mentioned in the other codes. Thus, overall, the participants had very positive opinions toward the usefulness of smartphone-based alcohol monitoring.

#### Overall opinions on technology used

The same positive features of the study were identified when participants were asked about their specific experiences while using the technology during the study. Four participants (20%) stated that the technology was either easy to use or was easy once they got used to it. For example, one participant said: “Once I got used to the iPhone, it was very easy. Now the breathalyzer, that was no problem, all you gotta do is just turn it on and blow. That’s simple.” Six participants (30%) described the various forms of technology as simply cool, interesting, fun, and/or effective, such as the individual who said “I thought it was cool, it was interesting.” Three individuals (15%) did mention having some technical difficulties, such as not being used to smartphones or connecting to service.

We also asked participants in a separate question if there was anything challenging associated with using the technology. Seven participants (35%) reported that there was nothing challenging related to using the technology, while one individual stated “It was challenging. But once I got the hang of it, it was ok”—suggesting that there was a bit of a learning curve associated with using the iPhone and breathalyzer. Six participants reported on specific challenges (30%), including difficulties with unlocking the phone (the phones were locked with a numeric passcode to protect privacy), turning on the breathalyzer, or cell phone service issues. One participant noted, “One time it wouldn’t come on and I think I was just exhausted and I realized I needed to charge it up. And I was like what is going on! And I realized I needed to charge it. But that wasn’t a huge problem.”

Finally, we asked if participants liked or disliked using technology to monitor their behaviors and why. No participants stated that they disliked using technology for this purpose. Thirteen participants (65%) stated that they generally liked or enjoyed the technology, saying things like “I liked it. I wish I could keep it.” Four individuals (20%) stated that they thought the technology was educational, informative, or could be useful for a wider audience. One individual stated “I’ve even thought that it eventually probably needs to be mobilized so that it’s a requirement for everybody” while another said “I like it because it gives you the reading right then on what you need to know.”

#### Feedback on text message reminders

In terms of feedback on the frequency of texts received over the course of the study, many participants (9/20, 45%) reported that being texted twice a day was fine and was not overburdensome. In fact, three participants stated that we could have texted more, with one participant stating that they sometimes missed the prompts. Two participants (10%) stated that they still sometimes forgot to complete their monitoring tasks even with the prompt, so they could have used a follow-up reminder. Overall, these findings indicate that the frequency of receiving two text messages a day was generally acceptable in this sample.

#### Feedback on the breathalyzer

We also asked participants to comment specifically on the BACTrack breathalyzer used in the study, asking about their overall experience and if they believed it was easy or difficult to use. The majority of participants (11/20, 55%) stated that the breathalyzer was easy to use. One participant said, “It wasn’t [difficult]. The device it walks you right through it.” Several participants (8/20, 40%) did report having occasional difficulties, however. These included difficulties with breathing long enough to get a reading, losing the plastic mouthpiece that connects to the breathalyzer (a small removable piece that was changed between research participants), or getting the breathalyzer to turn on. For example, one participant stated, “When you’ve had something to drink and you’ve been smoking and doing whatever and when you try and blow, that’s a lot of air you’ve got to blow into it!”.

## Discussion

Overall, results for the TRAC pilot study suggest that, despite positive attitudes toward the technologies, the feasibility of mobile monitoring of alcohol consumption using breathalyzers and mobile surveys remains questionable among PLWHA. Adherence rates for the breathalyzer readings ranged from 71 to 80%, depending on if you consider the two entirely non-adherent individuals. There were fewer responses to the mobile surveys, with adherence rates ranging from 56 to 62%. It is possible that this is because the mobile surveys required an additional step after the reading was completed. Participants may have simply forgotten to complete the survey or were unwilling or unable to take the extra time needed to do so. In the future, developers might consider creating an app integrating the breathalyzer readings with a short survey that becomes available upon completion of the reading. At the time of writing, the BACTrack app does include a space for indicating the number of drinks consumed and adding additional notes, but a feature allowing the inclusion of a survey would be especially helpful for PLWHA who want to monitor multiple health metrics.

Results also indicated that participants were much more likely to complete monitoring tasks in the daytime than they were in the evening. This is concerning, as individuals generally drink more in the evenings [24], so the collected data may not provide accurate information regarding actual consumption behaviors. As we plan for follow-up research to this pilot study, we will seek to increase response rates to evening monitoring tasks by exploring options including providing higher incentives for completing evening monitoring tasks or scheduling multiple monitoring tasks in the evening hours to increase the likelihood of getting a response.

An especially interesting finding was that rates of reported alcohol use were higher among the mobile surveys than the breathalyzers. This could be due to the fact that participants often reported alcohol use from several hours prior that no longer showed up on a breathalyzer reading. Given that alcohol leaves the system at a rate of 0.015 BAC/h, even someone at the typical legal limit of 0.08 BAC would have a 0.000 reading after just over 5 h [25]. The data suggest this might be what was occurring; In the instances in which the breathalyzer and the survey responses conflicted in terms of whether or not they indicated alcohol use, a strong majority were times in which participants reported drinking in the survey but did not have a positive breathalyzer reading. In these cases, respondents appeared to have a small amount of alcohol (one drink on average) several hours prior to completing the breathalyzer reading. Thus, it appears as though the surveys were capturing drinking occasions that the breathalyzers could not capture when used on just a twice-daily basis. This is not necessarily a weakness of breathalyzer-based monitoring, but shows the value of using both self-reports and biological indicators of alcohol consumption when relying on ecological momentary assessment [14, 26]. It also suggests that breathalyzer readings may need to happen more frequently than two times a day. Participants in our study indicated that two texts a day was an acceptable amount in the qualitative interviews, with some stating that we could have texted more. However, follow-up research is needed to see if additional breathalyzer readings and surveys are indeed feasible and acceptable given the lower adherence rates found in this study.

This study also attempted to assess preliminary validity of mobile monitoring by comparing alcohol consumption captured via breathalyzers and mobile surveys with self-reported behaviors from a baseline survey. The results of these correlational analyses were mixed. Overall frequency of alcohol consumption was not associated with the data captured via monitoring, which was surprising. However, frequency of binge drinking and AUDIT score were associated with monitoring data, such that those who reported higher levels of binge drinking and higher AUDIT scores had higher BAC levels and more days where they reported drinking. This suggests that mobile assessment of alcohol use may be especially beneficial for capturing behaviors among those who engage in problematic drinking, but may not be as useful for more casual drinkers. Given that most health promotion interventions using mobile monitoring will likely be targeting problematic drinkers, this is likely not a significant problem with the EMA approach tested here, but warrants further research.

Given the overall question of feasibility, the results of this study suggest that more work needs to be done to make mobile alcohol monitoring via breathalyzers and mobile surveys feasible for this population. While there is not a widely-accepted standard for determining whether individuals are compliant to EMA, several researchers have suggested a cut-off of 80% [27]. A recent meta-analysis of EMA approaches among substance users found a pooled compliance rate of 75% [27], suggesting that EMA approaches in general may need to be re-worked in order to lead to acceptable compliance among substance-using populations. It is surprising that compliance rates were not higher in this study, given the positive feedback provided by participants during their exit interviews. While interviewer effects may have led to more positive reports, participants generally reported liking the technologies and the majority reported that they were easy to use. However, there were instances where participants reported experiencing intermittent technical difficulties, such as losing the breathalyzer mouthpiece or not having cellular service. Additionally, some reported having difficulty breathing into the breathalyzer for long enough to complete a reading. These technical difficulties may have contributed to the lower adherence rates and demonstrate the need for giving further training, providing back-up mouthpieces to participants, and potentially screening for respiratory issues before entry into the study. Another barrier to EMA completion that participants reported was simply forgetting to complete the reading after they received the prompt. The TRAC Study, in an effort to reduce burden, did not send reminders to participants if they did not complete a reading, which could explain the lower adherence rates in this study. Previous studies that have yielded higher EMA adherence rates have utilized reminder messages if individuals did not complete their reading [16] or a chance to complete a “make-up” reading [17]. We will explore these methods of encouraging EMA completion in future stages of this research to increase adherence rates.

### Limitations

This pilot research included a small sample size of PLWHA in the Southeastern United States. Thus, the results are likely not representative of the entire PLWHA population and may not accurately reflect drinking behaviors. Also, there was no guarantee that the participants themselves completed the breathalyzer readings, which served as a limitation. However, there was little reason for deception by the participants, as their incentives were not based on whether or not they consumed alcohol. A future recommendation to avoid this limitation is to use video confirmation or location collection of each study participant, which would serve as proof of identity. Another limitation of this study is that participants were asked about whether they consumed alcohol “today” in the mobile surveys. It is possible that most participants assumed that meant since they woke up, which meant any alcohol consumption that occurred between the evening monitoring task and when participants woke up may not have been captured. Future research should seek to use more precise language to avoid any confusion among respondents. Finally, the small sample size, missing data, and limited time frame prevented the inclusion of more sophisticated data analyses. Moving forward, the next step of this research involves a longer monitoring period and a randomized controlled trial with a larger sample size to test the impact of monitoring in combination with an educational intervention.

## Conclusions

The TRAC pilot study represents an important first step toward examining the feasibility of smartphone-based monitoring of alcohol consumption among PLWHA. Overall, this study suggests that smartphone-based EMA combined with breathalyzers shows some promise for tracking alcohol consumption among this population, though improvements are needed to increase adherence to assessments, particularly for evening monitoring tasks. Results also indicate that this method of mobile monitoring may be especially valuable for collecting data regarding binge or hazardous drinking. Ongoing research will build on these findings and examine the value of monitoring in concert with an alcohol reduction intervention.

## Data Availability

The datasets generated and/or analyzed during the current study are available from the corresponding author on reasonable request.

## Abbreviations

AUDIT: alcohol use disorders identification test
BAC: blood alcohol content
EMA: ecological momentary assessment
PLWHA: people living with HIV/AIDS
RQ: research question
TRAC: tracking and reducing alcohol consumption
